# Successful Management of Persistent Gestational Trophoblastic Neoplasia: A Comprehensive Review and Case Analysis

**DOI:** 10.7759/cureus.51112

**Published:** 2023-12-26

**Authors:** Susmita Das, Madhusmita Sethy, Siddharth Sankar Das, Pawan Kumar J Maniyar

**Affiliations:** 1 Obstetrics and Gynaecology, Aster DM Hospital, Dubai, ARE; 2 Pathology, All India Institute of Medical Sciences, Bhubaneswar, Bhubaneswar, IND; 3 General Surgery, Dubai Hospital, Dubai, ARE; 4 Radiodiagnosis, Aster DM Hospital, Dubai, ARE

**Keywords:** human chorionic gonadotrophin, beta-hcg, molar pregnancy, hydatidiform moles, gestational trophoblastic neoplasia

## Abstract

Gestational trophoblastic disease comprises hydatidiform mole (HM) (complete or partial) and gestational trophoblastic neoplasia (GTN). Complete and partial moles have different karyotypes, gross and microscopic histopathology, clinical presentation, prognosis, and chances of progress to GTN. Ultrasonography (USG) and human chorionic gonadotropin (hCG) quantification are commonly used to diagnose molar pregnancy and further follow-up until resolution. Our case reports two patients, one with a complete mole and another with a partial mole, who were evaluated and followed up with serial beta hCG as per protocol and were found to have persistent disease and referred for chemotherapy until complete resolution. Fifteen to 20% of the patients with complete moles and about 1-5% of patients with partial moles developed GTN, which is primarily invasive. Hence, proper follow-up and chemotherapy assure 100% curability.

## Introduction

A hydatidiform mole (HM) is a pregnancy-related complication that comes under gestational trophoblastic disease, which also includes choriocarcinoma, placental site trophoblastic tumor, and invasive mole. An HM can be classified as complete or partial based on histopathology, ultrasound features, quantification of human chorionic gonadotropin (hCG), and other ancillary methods, such as flow cytometry.

Molar pregnancies are generally managed with an evacuation strategy, given that the patient has no accompanying medical complication [1]. Suction curettage is one of the safest methods of uterine evacuation due to the low risk of blood loss, infection, and retention of molar mass. Furthermore, this method does not compromise with future chances of conception. Hysterectomy may be performed in patients unwilling to conceive in the future or who have completed childbearing. In addition, about 18-28% and 2-4% of patients with complete and partial moles, respectively, develop persistent gestational trophoblastic neoplasia (GTN) [2]. Benign GTN is localized within the uterus, and there is no sign of the disease beyond it. At the same time, in metastatic GTN, the choriocarcinoma can spread well beyond the uterus to the pelvic region, vagina, and even to the lungs. In such conditions, monitoring of serum hCG becomes vital to predict the progression of GTN.

## Case presentation

We have two cases of molar pregnancy, one complete and the other partial, where after an uneventful evacuation, the hCG levels were persistent on monitoring and were managed by chemotherapy until resolution.

Case one

A 34-year-old female patient, married for seven years, attended OPD for infertility. She had a history of slightly prolonged cycles. Her hormonal profiling was conducted. Hypothyroid state and vitamin D deficiency were corrected. She was followed for one natural cycle and was found to have an unruptured follicle with low progesterone on day 21. 

**Figure 1:**
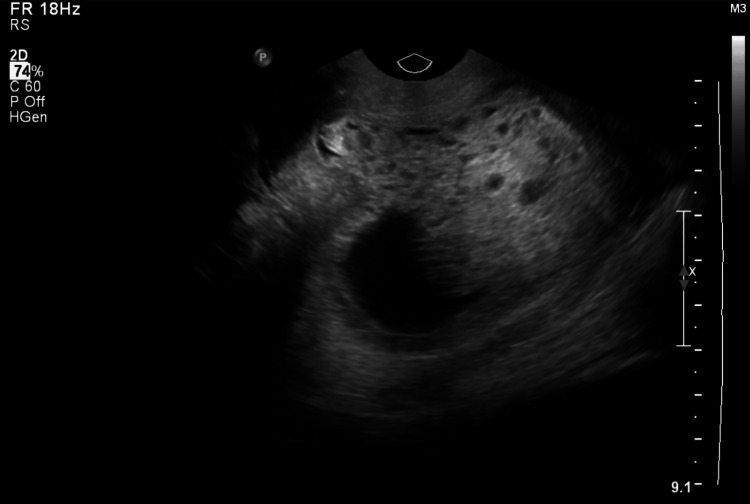
Ultrasound at nine weeks showed no fetal pole with complex echogenic intrauterine masses containing multiple cystic areas

She started with 100 mg clomiphene on the next cycle, and she became pregnant. An early follow-up scan showed no fetal pole at nine weeks with signs of molar features (Figure 1). Her β-hCG was elevated and was more than doubling. After that, she underwent evacuation at an initial β-hCG value of 4,138,515 mIU/mL. 

**Figure 2:**
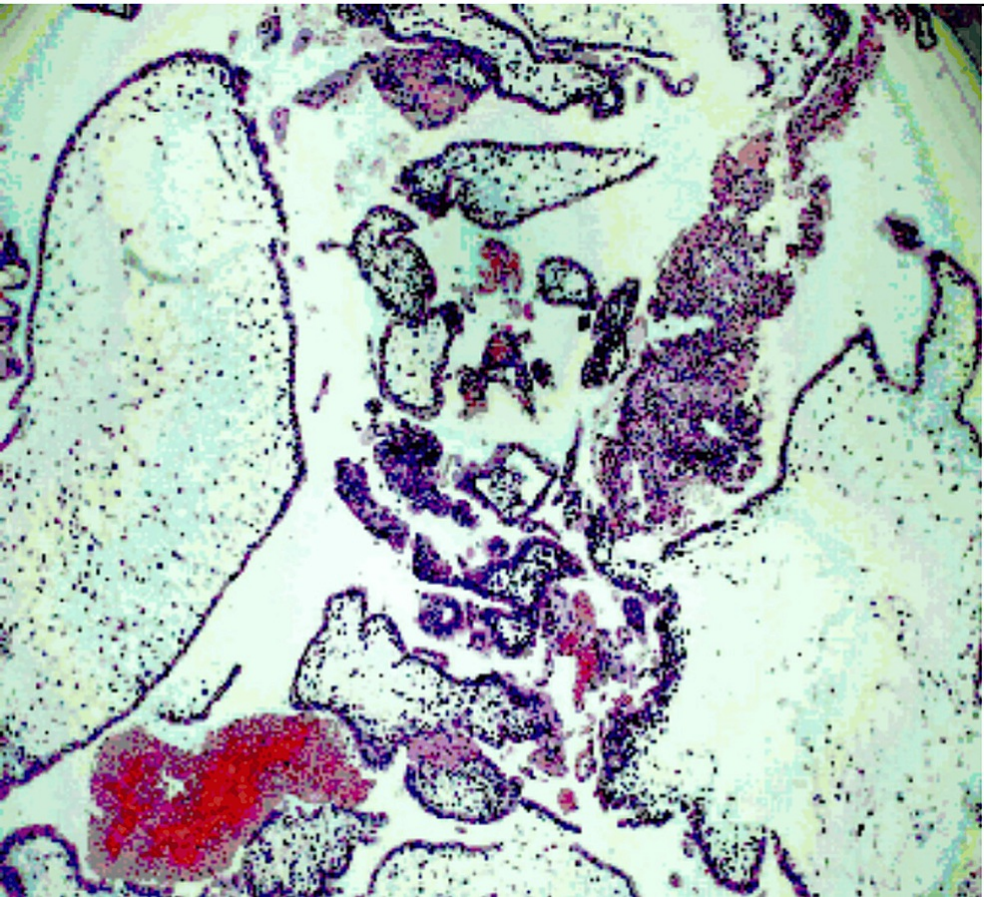
Microscopic features suggestive of a complete mole

Histopathology showed multiple chorionic villi of varying sizes with diffuse, circumferential trophoblastic (cytotrophoblast and syncytiotrophoblast) proliferation with avascular edematous villi; some of them show central cisterns. Accompanied are endometrial tissue with extensive decidualization; some show gestational hyperplasia. Haemorrhage and necrosis were also noticed in some areas. Features are of gestational trophoblastic disease, suggestive of a complete mole (Figure 2).

Post evacuation, she was advised to follow up with weekly β-hCG evaluation. It was observed that the β-hCG levels of the patient had started to plateau between the third and sixth weeks. She reported recurrent bleeding and hence was advised for a repeat sonography scan and X-ray. The scan did not indicate any retained molar tissues in view of persistent GTN disease. She was then referred to the medical oncology department for chemotherapy. She received five cycles of methotrexate, with each cycle lasting for one week. Her follow-up β-hCG was negative for six consecutive months after her last cycle.

Case two

A 38-year-old female patient, para two living two, with two previous caesarean sections, was referred to the doctor’s clinic at 14 weeks with on and off vaginal spotting and a scan report suggestive of molar pregnancy. She was hypothyroid on 125 mcg Thyronorm and had no other medical comorbidities. 

**Figure 3:**
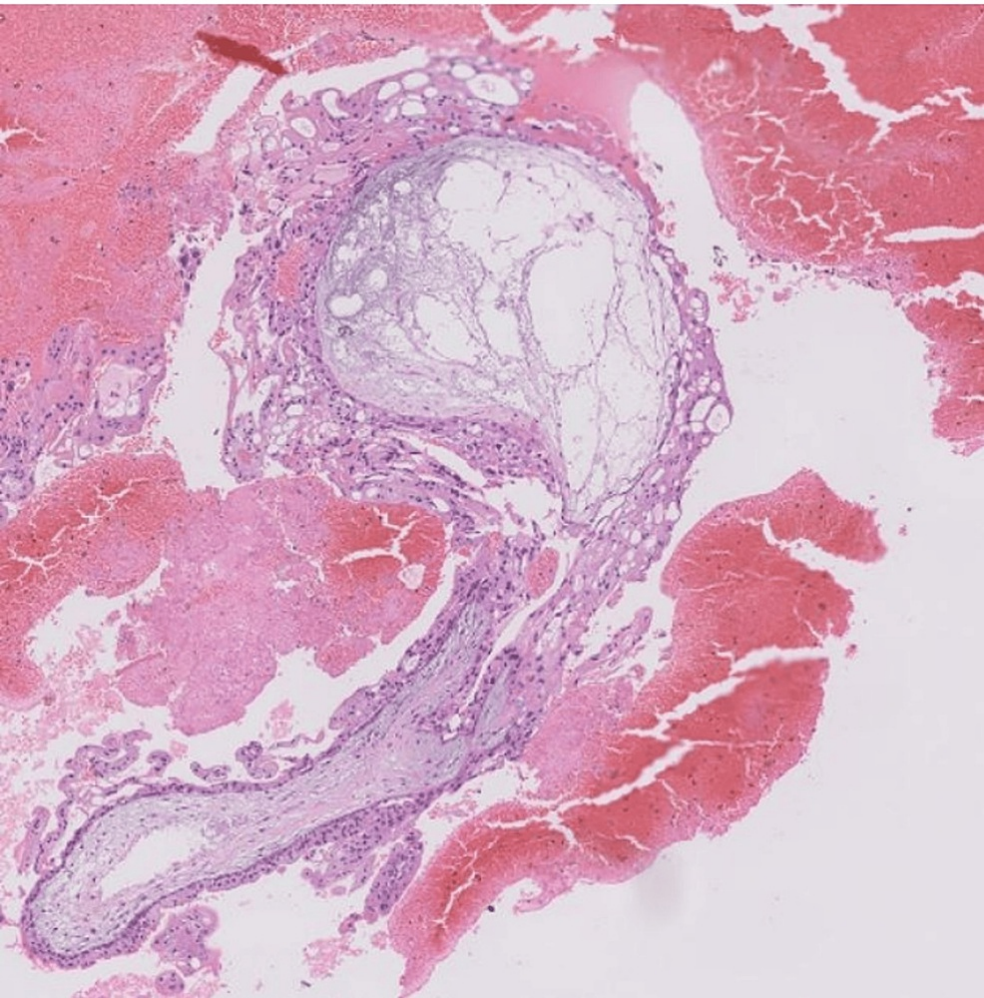
Histopathology features suggestive of a partial mole.

Her last caesarean was five years back. She was planned for dilatation and evacuation under general anaesthesia. Her initial β-hCG level was 197,233 mIU/mL. She was evacuated to the OT with oxytocin and under scan guidance, with an estimated blood loss of 1,300 ml. Her repeated β-hCG value was 53,245 mIU/mL the next day. Her histopathology report showed large fragments of inflamed and avascular decidua, along with multiple infarcted hyalinized and few viable chorionic villi. Few villi showed an extensive trophoblastic proliferation, with hydropic changes and central avascular cisternae formation, features suggesting a partial HM (Figure 3).

She was advised for weekly evaluation of β-hCG levels since she had a history of recurrent bleeding during follow-up with rising β-hCG values. Her repeat pelvic scan revealed an ill-defined heterogeneous area measuring 9.6 x 4.7 mm. The patient had a CT scan of the abdomen chest X-ray, and no metastasis was found. She was then referred to the medical oncology department and received chemotherapy.

## Discussion

With better diagnostic methods, such as ultrasonography and sensitive β-hCG assays, it has been possible to detect HM in early pregnancy [3]. Ngan et al. (2018) reported that complications in HM occurred in 25% of patients with an enlarged uterus bigger than that during 14-16 gestational weeks [4]. After a complete mole, 18-28% of patients develop GTN. An invasive disease is several fold more common in these cases than a metastatic disease. After a partial mole, 2-4% of patients develop GTN. Almost all patients have invasive diseases in such cases, while metastatic diseases are rare [2]. In their study, Sun et al. reported that even though early diagnosis and evacuation were possible, they did not affect the appearance of post-molar GTN in the patients [5]. 

**Figure 4:**
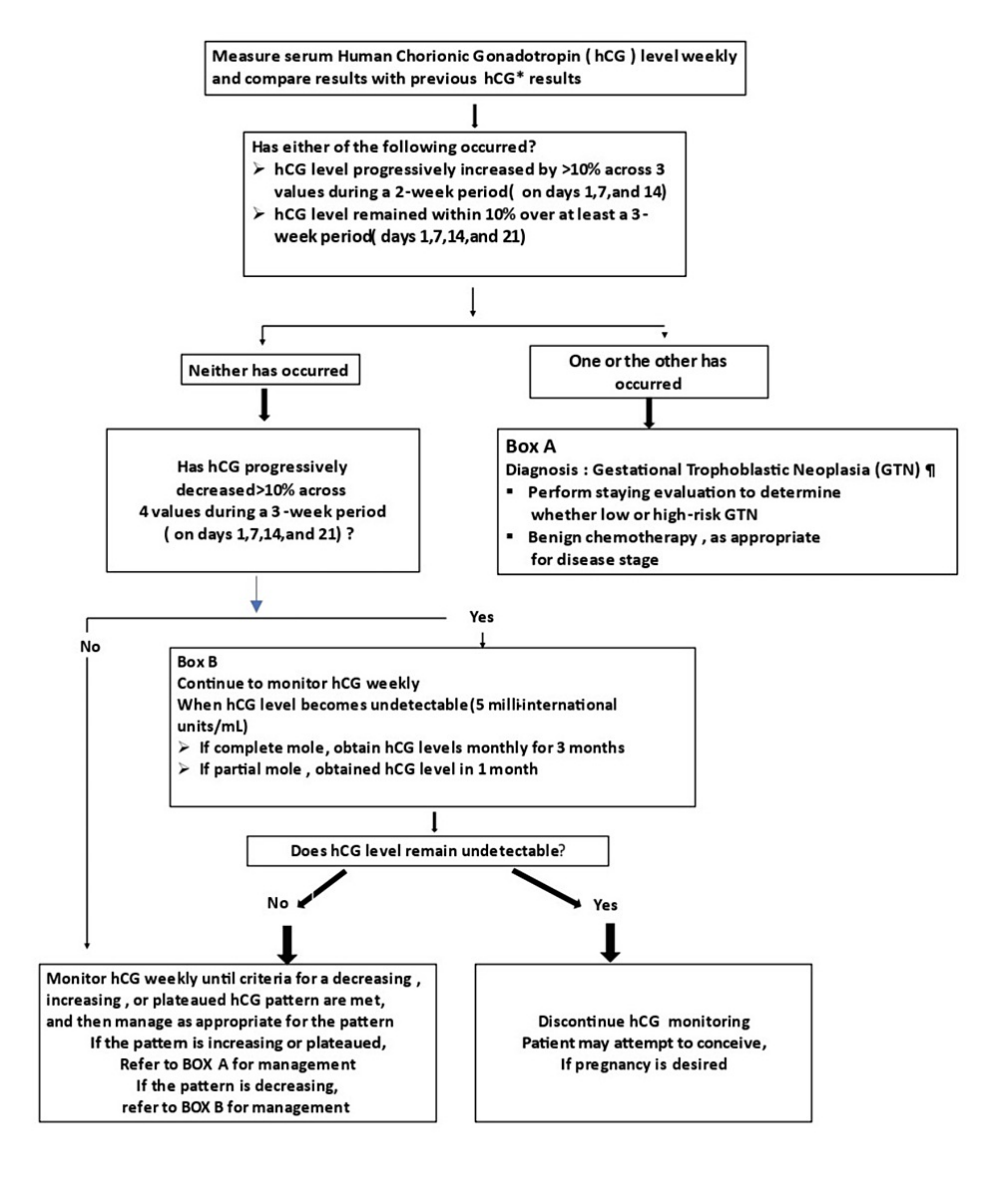
Algorithm monitoring after surgical treatment of the hydatidiform mole hCG: human chorionic gonadotropin, GTN: gestational trophoblastic neoplasia

The progression of HM into persistent GTN can be monitored with quantitative serum β-hCG evaluation. After the surgical evacuation or hysterectomy, β-hCG is measured every week until it is undetectable or the criteria for increased or plateaued level are met, as per the algorithm (Figure 4) [6]. Commercially available assays can measure β-hCG to its baseline values (<5 mIU/ml) [7]. 

With a post-evacuation hCG regression curve, Sasaki (2003) analyzed that in a spontaneous resolution, hCG levels dropped below 1,000 mIU/ml within five weeks of initial evacuation [8]. However, with the progression of persistent GTN, the hCG titre remained above 1,000 mIU/ml even after five weeks. Likewise, around eight weeks, hCG levels were below 100 mIU/ml in patients with spontaneous disease resolution. In patients who developed GTN, hCG titres were found elevated even around 20 weeks post-evacuation. Sasaki concluded that hCG levels were well below the detectable limit within 20 weeks in spontaneous resolution. 

Van Trommel et al. (2005) showed that evaluation of different forms of hCG is required for precise monitoring of HM progression [9]. A plateaued or rising level indicates GTN and is treated with chemotherapy later. A decreasing and undetectable hCG level is defined as a level that progressively decreases more than 10% across four values in three three-week periods. Once the β-hCG level becomes less than 5 mIU/mL, further monitoring is done for three months for complete moles and one month for partial moles with negative values.

Furthermore, Ngan et al. (2018) recommended that patients with GTN should be monitored for all forms of hCG as the neoplasm tissues tend to secrete abnormal forms of hCG [4]. Coyle et al. (2018) studied 20,000 women in the UK who had undergone HM evacuation [10]. They found that the risk of developing post-molar GTN in patients with partial moles was 1:3,195, and with complete moles, it was 1:406. The risk of GTN after normalization of hCG was 3.8 times higher with complete moles. They suggested that patients with complete moles should be monitored for elevated hCG for up to six months post-evacuation. Herein, hCG levels of the patient with complete moles plateaued for three to six weeks, after which GTN was suspected. In the other case with partial moles, the hCG levels of the patient rose post-evacuation, indicative of GTN. Both patients received chemotherapy after the diagnosis of GTN.

Patients with molar pregnancy are always advised to use reliable contraception during the entire interval of hCG monitoring because a new pregnancy event during this period makes it difficult to interpret the hCG results and complicates the management process. Randomized studies performed by several groups demonstrated that moderate to low doses of oral contraceptives (OCs) do not increase the occurrence of post-molar GTN but significantly prevent pregnancies during post-molar monitoring [11,12]. Therefore, combined OC or progestin-only pills or barrier methods are advised during the monitoring phase. Pregnancy can be encouraged only after completely ruling out GTN. Patients with a prior HM have a 1-2% chance of getting a second mole in the subsequent pregnancy [13]. Therefore, all further pregnancies should be accompanied by ultrasonographic examination as early as the first trimester. Genetic mutations in NLRP7 and KHDC3L have been identified in many women with HM, and hence, women with consecutive molar pregnancies must undergo germline testing of these genes. The following are some risk factors for post-molar GTN: women older than 40 years, women of Asian origin, pre-evacuation hCG levels above 100,000 mIU/ml, excessive enlargement of the uterus, or theca lutein cysts larger than 6 cm [14].

Patients with GTN present high remission rates when treated with chemotherapeutic agents, such as methotrexate and actinomycin D [15]. Wang et al. (2017) recommended that prophylactic chemotherapy accompanied with short methotrexate or actinomycin D regimens decreased the incidence of post-molar GTN as compared to patients who did not receive any chemotherapy (P < 0.001) [16]. A trial compared the effectiveness of intramuscular methotrexate with bolus intravenous actinomycin D [17]. Among the 216 women, bolus actinomycin D showed a better primary remission rate than methotrexate (70% vs. 53%, P = 0.01). Serum hCG monitoring during chemotherapy provides insight into the effectiveness of the treatment, and consolidation therapy is continued till hCG levels normalize. Many studies have shown that consolidation chemotherapy of one to three cycles presented relapse rates as low as 0-8.4% [17-19]. A patient under chemotherapy is advised to avoid pregnancy for at least one year post-treatment.

## Conclusions

After evacuation of molar pregnancy, trophoblastic tissue can persist in up to 20% of cases. Surveillance is essential after evacuation to exclude the presence of persistent diseases. The diagnosis is based on finding the stable or serially rising serum β-hCG rather than examination of tissue. Thus, patients are treated empirically with chemotherapy accordingly. Due to their rising trend of β-hCG levels, they usually get referred to medical oncology and receive chemotherapy on follow-up visits.
